# The NIDA clinical trials network: evolving, expanding, and addressing the opioid epidemic

**DOI:** 10.1186/s13722-021-00238-6

**Published:** 2021-05-08

**Authors:** Betty Tai, Ronald Dobbins, Quandra Blackeney, David Liu, Landhing Moran

**Affiliations:** Center for the Clinical Trials Network, National Institute On Drug Abuse, National Institutes of Health, 3WFN Room 09A48 MSC 6022, 301 North Stonestreet Avenue, Bethesda, MD 20892 USA

**Keywords:** National Drug Abuse Treatment Clinical Trials Network, Opioid use disorder, HEAL, Substance use disorder treatment

## Abstract

Over the past two decades, the National Drug Abuse Treatment Clinical Trials Network (CTN), a program of the National Institute on Drug Abuse (NIDA), has expanded from the initial six Nodes to 16 Nodes, as a nationwide consortium of research scientists and treatment providers working together to improve care for substance use in the nation’s communities. Encompassing both specialty care programs and general medical settings, the Network has become a unique resource for expertise on clinically focused research, bridging the gap between research and treatment delivery. Over 22 years, the CTN has completed 101 studies, resulting in 650 publications. In response to the opioid epidemic, a CTN task force generated a comprehensive list of research priorities in the areas of prevention, treatment, knowledge dissemination, and workforce training, to form the basis of the Network’s opioid portfolio. The Network’s opioid portfolio currently includes five main categories of studies: (1) large multi-site studies; (2) studies aimed at closing the treatment gap; (3) expansion of ongoing studies to improve service delivery and implementation; (4) studies to explore the use of substance use data in electronic health record systems; (5) training and dissemination projects to expand the research/health care provider workforce. With funding from the Helping to End Addiction Long-Term Initiative^SM^ (HEAL), the CTN established five new Nodes, which, along with the pre-existing Nodes, are distributed in every region of the nation and engage researchers and clinicians in areas that have been among the hardest hit by the opioid epidemic. Through this expanded network and its commitment to developing personalized, evidence-based treatments, the CTN is poised to address and provide solutions for the ongoing epidemic of opioid use and addiction.

## Background

The National Drug Abuse Treatment Clinical Trials Network (CTN), a program of the National Institute on Drug Abuse (NIDA), is a nationwide consortium of research scientists and treatment providers working together to improve care for substance use in the nation’s communities. NIDA established the CTN in 1999 following an Institute of Medicine (IOM) call for academic researchers to focus greater attention on treatment providers’ needs, and for providers to more readily adopt evidence-based practices [1]. Over the course of two decades, the Network has become a unique resource for expertise on clinically focused research with an extensive infrastructure that encompasses both specialty care programs and general medical settings. Today, it is ideally positioned to rapidly test and disseminate practical, readily applicable solutions to alleviate the nation’s deadly epidemic of opioid use and addiction.

### At the interface of science and treatment provision

The CTN is a Network of Nodes, each comprising one or more academic centers linked to nearby health systems, treatment programs, and provider practices. The researchers and providers in the CTN Nodes collaborate to design and conduct rigorous, multi-site clinical trials and other clinical studies that have potential to improve substance use treatment access or outcomes. To date, the Network has initiated over 100 studies, and Network investigators have published over 650 papers. This work has opened pathways for significant new treatment approaches and shed important light on treatment alternatives. One recent trial, for example, was instrumental in convincing the U.S. Food and Drug Administration to grant clearance, for the first time for any disease, for a digital therapeutic [2]. The trial showed that the therapy, called reSET in its commercial digital therapeutic form, effectively treats cannabis, cocaine, stimulant, and alcohol use disorders [3].

To initiate a research project in the CTN, the researchers and providers in a Node or Nodes submit a research proposal to the CTN Research Development Committee for peer review. Review criteria are no less stringent than those used to assess the worthiness of any NIH research: (1) significance of the research question, (2) investigators’ experience, (3) innovation, (4) approach to the research question, (5) environment, (6) readiness for multi-site implementation, and (7) budget. Proposals with adequate scores and programmatic priority advance to research protocol development, with input and assistance from the Network data and statistics coordinating center and clinical coordinating center. Most projects in the CTN are led cooperatively by investigators from multiple Nodes; this practice, and the multi-site nature of the trials, ensure that as many collaborators as is practical feel ownership in, contribute to, and learn from the project.

Through their participation in the CTN, researchers have become better acquainted with treatment providers’ practical concerns, and providers have learned to formulate research questions and conduct clinical research with high scientific and ethical integrity. Young scientists also obtain training and mentoring from the CTN, either by joining a project or through a fellowship arrangement. The CTN’s training platform also extends internationally, via its INVEST fellowships.

For the first years of the Network’s existence, almost all participating treatment providers worked in specialized community substance use treatment programs. Today, partners include primary care, emergency care, and other non-substance use specialty providers. This crucial expansion enables the Network to take advantage of opportunities arising from health care reforms that have integrated substance use care, for the first time, with mainstream medicine [4]. The Mental Health Parity and Addiction Equity Act of 2008 and the Affordable Care Act (ACA) of 2010 mandate that insurers cover and reimburse treatment for substance use disorders similarly to other chronic diseases [5]. Federal and state laws now authorize practitioners with a broader array of credentials to provide substance use treatment, and in more diverse settings, compared to previously.

These reforms established essential conditions for the goal that has guided CTN research since its beginning: a comprehensive treatment system that answers to the chronic nature of substance use disorders [6]. Such a system will integrate substance use treatment with care for comorbid somatic and psychological conditions. In such a system, every encounter with a medical professional will include an assessment potentially leading to treatment or referral for treatment. However, although health care reforms have greatly enhanced prospects for reaching this goal, many challenges remain in the way of making it a reality. These include identifying appropriate roles for many types of practitioners in substance use treatment, assessing and addressing their educational needs with respect to substance use issues, and establishing mechanisms of coordination. The CTN, with its abundant experience in practice-oriented research and rich two-way relationships with providers in both specialty care and general medical settings, is well equipped to tackle these problems.

### The Network and the opioid epidemic

In 2015, after rising steadily since 1999, the number of people in the U.S. dying annually from opioid overdoses began a sharp ascent [7]. By 2017, the yearly death toll was over 47,000 [8], more than the number of traffic fatalities [9], and an estimated 2.1 million people were estimated to have an opioid use disorder [10]. For the first time in decades, overall life expectancy in the United States fell, due in large part to deaths from drug overdoses [11].

Alarmed by this escalation, the CTN in 2016 formed a special opioid task force to determine how best to mobilize its resources to combat the epidemic. The task force generated a comprehensive list of research priorities in the areas of prevention, treatment, knowledge dissemination, and workforce training (Table 1). This list remains the basis of the Network’s opioid research portfolio today.

**Table 1:** CTN Opioid Research Goals

Prevention
•Develop brief, sensitive screening tools suitable for use in busy medical practices
•Detect risk levels for opioid misuse and abuse
•Match risk levels to appropriate actions, e.g., counseling or treatment
Treatment
•Increase access to treatment
•Expand patient entry points to include, for example, ED, OB/GYN, neonatal care, pediatrics, infectious disease care, dental, pain clinics, criminal justice systems
•Engage community pharmacies as potential sites for patient recruitment
•Adopt effective methods used in other therapeutic areas to reach patients who are in resource-deprived or rural areas
•Improve OUD treatment quality
•Implement best practices of MOUD in specialty care and primary care settings
•Adopt chronic care model with emphasis on patient-centered approaches for OUD management
•Adapt digital technology such as eHealth to reach patients in rural areas; adopt electronic devices to enhance patient engagement, monitoring, and diagnosis
Dissemination
•Adapt the “learning health care system”
•Use electronic health record systems to engage providers and patients to participate in research and expeditiously translate research results into effective care
Training
•Train research workforce

The Network’s strategy assigns the highest priority to research and education to optimize the use of medication for opioid use disorder (MOUD). In the United States, medications approved for the treatment of opioid use disorder (OUD) include buprenorphine, extended-release naltrexone, and methadone; buprenorphine and extended-release naltrexone can be prescribed and managed in office-based practice [12, 13]. If used to their fullest potential, these medications could significantly curtail the impact of the opioid epidemic. Instead, they are grossly underused [14–16], and when they are used, it is often to less than their full potential [17]. Multiple factors contribute to this situation, including regulations that require special licensing and limit caseloads, lack of proficiency in initiating and managing these treatments, and disjointed service delivery and coordination [18, 19]. Other barriers include limited geographic access to treatment providers [20], stigma associated with OUD, and a lack of resources for many patients who are in compromised economic conditions [21].

The CTN set objectives of increasing MOUD use in general medical settings and improving MOUD treatment quality and patient engagement, all within the context of a fully integrated continuum of care. A recent multi-site trial illustrates the CTN’s capacity to promote these goals by clarifying issues that can complicate and hinder effective treatment decisions. The results demonstrated that buprenorphine and extended-release naltrexone are equally effective for patients who complete initiation, but that more patients drop out of treatment before completing initiation on extended-release naltrexone [22]. This is highly useful information for providers and patients; it reinforces the need to assist patients through the difficult process of completely withdrawing from misused opioids that must precede initiation on naltrexone, an opioid antagonist. (Buprenorphine, an opioid partial agonist, requires only that patients enter withdrawal.)

In 2017, the president declared the opioid epidemic a national public health emergency [23]. As part of the government-wide response to the epidemic, Congress gave the National Institutes of Health (NIH) a supplemental appropriation to conduct the Helping to End Addiction Long-Term Initiative^SM^ (HEAL) (https://heal.nih.gov/). The NIH and NIDA directed a substantial share of HEAL funding to an expansion of the CTN and its opioid research efforts, recognizing the Network’s mature capacity and readiness to rapidly test ideas for practice improvements, in trials ranging from small and local to nationwide, and to facilitate the introduction of research findings into actual practice.

**Fig. 1:**
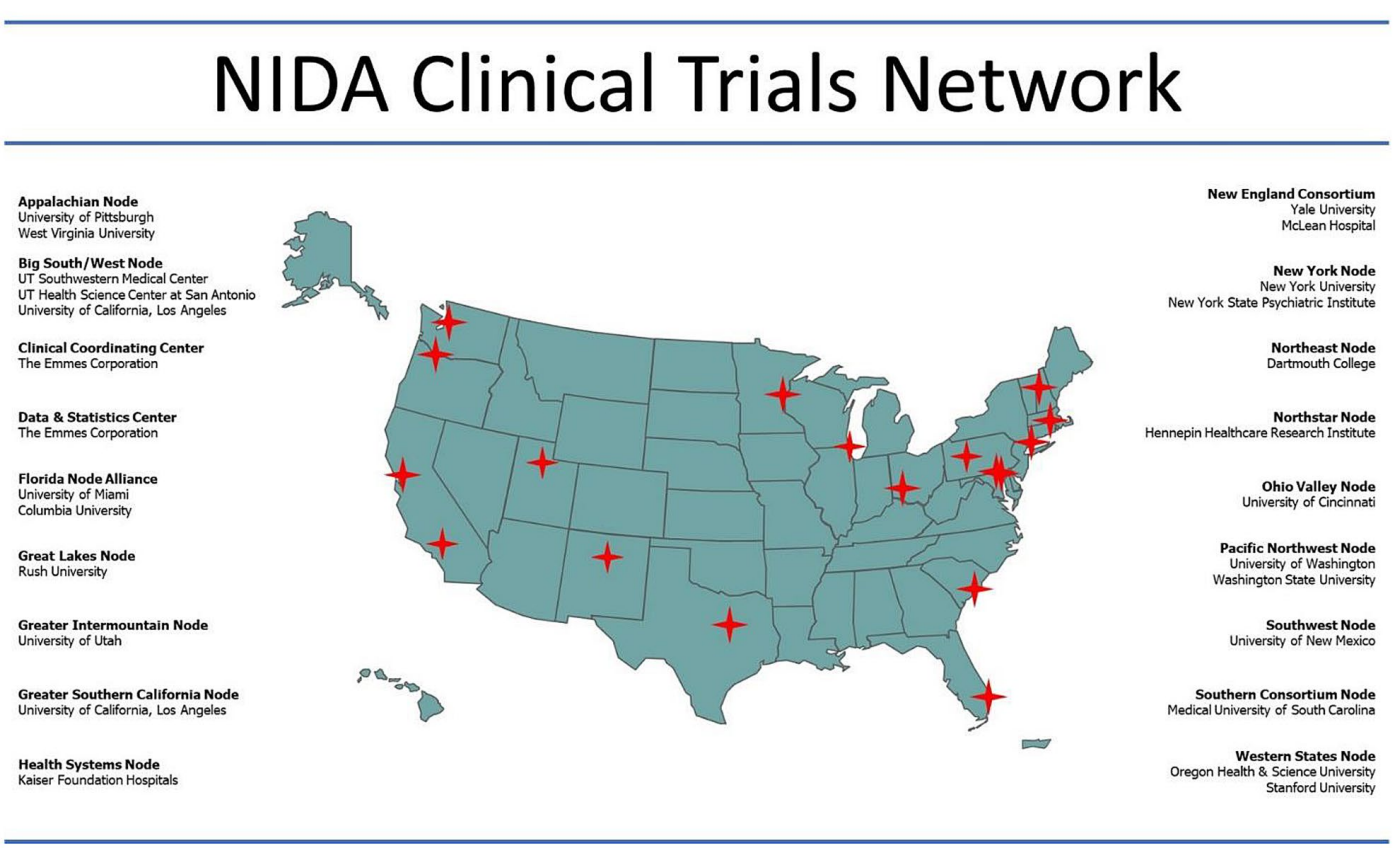
Map of CTN Nodes

With HEAL funding, the CTN established five new Nodes, which, along with the pre-existing Nodes, are distributed in every region of the nation (Fig. 1). The new Nodes have engaged researchers and clinicians in areas that have been among the hardest hit by the opioid epidemic: Appalachian (Western Pennsylvania and West Virginia), Greater Intermountain (Utah), Great Lakes (Illinois and Wisconsin), Southwest (New Mexico), and Greater Southern California. The Network expanded its already ambitious opioid portfolio to encompass five categories of studies (Table 2):

- Large multi-site studies to answer questions that previously were out of reach due to budget constraints. Among these are: (a) how best to engage and equip personnel at more patient encounter points, (e.g., family medical practices, community pharmacies, obstetrics and gynecology practices) to identify and refer people with substance use issues for treatment, etc.; (b) how to efficiently coordinate substance use treatment with treatment for other patient conditions in primary care; and, (c) how to amplify the substance use treatment workforce.
- Studies aimed at closing the treatment gap, by determining how best to deliver quality treatment to severely underserved populations.
- Expansion of ongoing studies to efficiently generate essential knowledge on improving service delivery and implementation.
- Studies to explore the inclusion of substance use data in electronic health record systems, and the potential for these data to generate relevant clinical information and enhance patient care.
- Training and dissemination projects to expand the research/health care provider workforce.

In recognition of the dissemination and implementation hurdles that often slow the translation of research findings into practice, many of these studies are designed to evaluate not only the clinical effectiveness of the treatment strategies of interest, but also the feasibility and efficacy of methods for implementing those strategies in the “real world.”

**Table 2:** CTN HEAL Projects

CTN No	Project title			Lead Investigators (Node)		Design		Outcomes		Settings	# of Sites (N)
Large, Multi-Site OUD Trials											
CTN-0080	Medication Treatment for Opioid Use Disorder in Expectant Mothers (MOMs): A Pragmatic Randomized Trial Comparing Extended-Release and Daily Buprenorphine Formulations		Theresa Winhusen (Ohio Valley)			2-arm RCT (SL-BUP vs. XR-BUP)		Illicit opioid use during pregnancy and 12-month postpartum; NOWS severity		Office-based	12 (300)
CTN-0097	Surmounting Withdrawal to Initiate Fast Treatment with Naltrexone (SWIFT): Improving the Real-World Effectiveness of Injection Naltrexone for Opioid Use Disorder		Adam Bisaga, Ned Nunes (Greater New York)			Cluster randomized trial, 2 arms (rapid vs. standard XR-NTX induction)		Proportion of patients receiving first XR-NTX injection		Specialty care	6 (Up to 450)
CTN-0098	Exemplar Hospital Initiation Trial to Enhance Treatment Engagement (EXHIT ENTRE)		Gavin Bart (Northstar) Todd Korthuis (Western States)			Comparative effectiveness trial, 2 arms (Addiction-Medicine Consultation Services (ACS) with XR-BUP vs. ACS TAU); Implementation trial		Proportion of patients engaged in OUD care on 34^th^ day following hospital discharge		Hospital	5 (314)
CTN-0099	Emergency Department-INitiated bupreNOrphine and VAlidaTIOn Network Trial (ED-INNOVATION)		Gail D’Onofrio, David Fiellin (New England Consortium)			2 arm RCT (SL-BUP vs. XR-BUP)		Effectiveness of XR-BUP and SL-BUP induction in ED on engagement in formal addiction treatment at 7 days		Emergency Department RCT	30 (2000)
CTN-0100	Optimizing Retention, Duration and Discontinuation Strategies for Opioid Use Disorder Pharmacotherapy		John Rotrosen, Ned Nunes (Greater New York) Roger Weiss (New England Consortium)			Retention trial with BUP: 2 behavioral interventions (TAU and TAU + reSET-O) × 3 meds (standard SL-BUP, high SL-BUP, XR-BUP); Retention trial with XR-NTX (TAU vs. TAU + reSET-O); Discontinuation trial for SL-BUP ppts: 2 (TAU and TAU + ACHESS) × 2 (SL-BUP taper and Transition to XR-BUP)		Retention in MOUD treatment at 6 months, 1 year, and 2 years; Successful taper within 1 year and no relapse for 6 months post-taper		Specialty care Primary care	20 (Retention, N = 1630; Discontinuation, N = up to 1000)
CTN-0101	Subthreshold Opioid Use Disorder Prevention (STOP) Trial		Jennifer McNeely (Greater New York) Jane Liebschutz (Appalachian)			Cluster randomized trial, 2 arms (STOP intervention vs. enhanced usual care)		Days of “risky” opioid use in adult primary care patients over 6 months of follow-up		Primary Care	5 (60 PCPs; 480 patients)
CTN-0102	Rural Expansion of Medication Treatment for Opioid Use Disorder		Yih-Ing Hser (Greater Southern California)			Feasibility trial; Pragmatic trial, 2 arms (OBOT vs. OBOT + telemedicine)		Proportion of OUD patients initiating or receiving MOUD and retention on MOUD in rural communities		Rural Primary care Telemedicine office-based opioid Treatment	40 (Phase I; N = 48,000); 30 (Phase II; N = 240,000)
Closing the Treatment Gap											
CTN-0088	DC Research Infrastructure Building & Initiative to Reach, Engage, and Retain in MAT patients with OUD		Richard Schottenfeld (Mid-Atlantic)				Single-arm: collaborative care model of MOUD provision; community-based outreach, engagement, and recovery support interventions		Treatment retention, adherence, and satisfaction	Specialty care	1 (170)
CTN-0093	Validation of a Community Pharmacy-based Prescription Drug Monitoring Program Risk Screening Tool (PHARMSCREEN)		Gerald Cochran (Greater Intermountain) Theresa Winhusen (Ohio Valley)				Cross-sectional, self-administered health survey		Substance use, risk for adverse opioid-related outcomes	Community pharmacy	15 (1523)
CTN-0095	Clinic-Randomized Trial of Clinical Decision Support for Opioid Use Disorders in Medical Settings		Rebecca Rossom, Gavin Bart (Northstar)				2-arm: OUD Clinical Decision Support or Usual Care		OUD diagnosis, Naloxone prescription, MOUD orders or referrals, total days covered by MOUD prescription	Primary care	30 (1500)
CTN-0096	Culturally Centered MAT for OUD Implementation Facilitation for Primary Care and Addiction Treatment Programs Serving American Indian/Alaska Natives		Kamilla Venner (Southwest) Aimee Campbell (Greater New York)				Cluster-randomized stepped wedge, 2-phase development and testing of MOUD implementation intervention		Proportion of OUD patients initiated on MOUD	Primary care Specialty care	4 (200)
CTN-0105	Integrating pharmacy-based prevention and treatment of opioid and other substance use disorders: A survey of pharmacists and stakeholders (Pharm-Serve-SUD)		Li-Tzy Wu (Mid-Southern)				Cross-sectional, one-time survey		Knowledge of, attitudes about, and intention to provide patient care and services for SBIRT for MOUD	Community pharmacy	1062 licensed community pharmacists recruited from pharmacist associations or pharmacy networks in U.S
CTN-0107	Peer recovery Support: A Bridge to Treatment for Overdose Survivors		Kelly Barth (Southern Consortium)				2-arm (Peer Recovery Coaches vs. TAU)		Engagement in formal OUD treatment, retention in treatment, number of overdoses after enrollment	Emergency department	3
Expansion of Existing OUD Studies											
CTN-0074-A-1		Primary care Opioid Use Disorder Treatment PROUD Economic Ancillary Study			Katharine Bradley Sean Murphy (Health Systems)		Observational (data collected from randomized controlled trial comparing collaborative care model and usual primary care for OUD)		Costs of collaborative care model intervention compared to usual care and net monetary benefit from healthcare sector perspective	Primary care	12 (~ 120,000)
CTN-0079-A-1		Ancillary Study of the Adoption and Sustainability of ED-Initiated Buprenorphine			Ryan McCormack John Rotrosen (Greater New York)		Implementation and feasibility study		Patients assessed, treated, and engaged in treatment; Factors influencing diffusion and effectiveness of clinical protocols for OUD screening, Bup treatment and referral	Emergency departments	2 (80–120)
CTN-0084-A-1		Determining the Optimal Duration of Buprenorphine Treatment to Reduce the Risk of Relapse, Overdose, and Mortality			Cynthia Campbell (Health Systems)		Observational		Risk of relapse, overdose, and mortality	Health systems EHR (registry)	10 health systems (1.8 M)

CTN No		Project Title		Lead Investigators (Node)	Database Source			
Database Studies								
CTN-0084	Developing a Prescription Opioid Registry across Diverse Health Systems		Cynthia Campbell (Health Systems)		Health Care Systems Research Network (HCSRN) Virtual Data Warehouse (VDW)			
CTN-0085	Selection Bias-Free Estimation of the Impact of Drug-Focused 12-step Mutual Help Groups		Keith Humphreys (Western States)		7 federally-funded randomized clinical trials of Drug Focused 12-Step Groups (DTSG) facilitation interventions			
CTN-0086	Inpatient versus Outpatient Treatment Outcomes for People with Opioid Use Disorder		Daniel Hartung (Western States)		Medicaid, Treatment Episodes Data Set (TEDS), and Vital Statistics data in Oregon and Oklahoma			
CTN-0087	A Foundation to Examine Reasons for Discontinuation for Buprenorphine Care in the Veterans Health Administration		Adam Gordon (Greater Intermountain)		VA Corporate Data Warehouse (CDW)			
CTN-0094	Individual Level Predictive Modeling of Opioid Use Disorder Treatment Outcome		Sean X. Luo (Greater New York) Daniel J. Feaster (Florida)		CTN Data Share			
CTN-0106	Derivation and Validation of New Measurement-Based Care Tools Derived from the Brief Addiction Monitor (BAM)		Andrew Saxon James McKay (Pacific Northwest)		VA Corporate Data Warehouse (CDW)			

CTN No	Project Title	Lead Investigators (Node)				
Training and Dissemination Studies						
CTN-0089	Integrating Nurse Practitioner Buprenorphine Wavier Training into Graduate Nursing Curriculum	Karen Hartwell Kathleen Brady (Southern Consortium)				
CTN-0090	Innovatively Increasing PCP Prescribing of Buprenorphine: Measurement Based Care and Integrated Electronic Solution (MBC4OUD)	Adriane dela Cruz Madhukar Trivedi (Texas)				
CTN-0091	Preventing and Identifying Opioid Use Disorder using the Six Building Blocks (6BBs) for Improving Opioid Prescription Management	Laura-Mae Baldwin (Pacific Northwest)				
CTN-0103	Expanding Clinical Research Training on Implementing the Evidence-based Hub and Spoke Model of Medication-Assisted Treatment for Opioid Use Disorder	Lisa Marsch (Northeast Node)				

## Conclusion

The NIDA CTN, like the IOM, views bridging research and treatment provision as a critical route to a treatment system that can provide every patient with a personalized, evidence-based continuum of care for their substance use disorders. The Network has nurtured and continues to expand a nationwide contingent of clinically aware researchers, clinician-researchers, and research-attuned providers. Throughout its history, the Network has adapted to take advantage of new opportunities, recent examples being research on digital therapeutics [24] and data science [25]. Its history and growth have endowed the CTN with capacity to play a unique role in addressing the ongoing epidemic of opioid use and overdose.

## Data Availability

Not applicable.

## Abbreviations

CTN: Clinical Trials Network
NIDA: National Institute on Drug Abuse
IOM: Institute of Medicine
ACA: Affordable Care Act
MOUD: Medication for Opioid Use Disorder
OUD: Opioid Use Disorder
NIH: National Institutes of Health
HEAL: Helping to End Addiction Long-term
NOWS: Neonatal opioid withdrawal syndrome
